# Editorial: Carbohydrate-Restricted Nutrition and Diabetes Mellitus

**DOI:** 10.3389/fnut.2021.827990

**Published:** 2022-01-21

**Authors:** Eric C. Westman

**Affiliations:** Department of Medicine, Duke University Medical Center, Duke University, Durham, NC, United States

**Keywords:** insulin resistance, type 2 diabetes, metabolic syndrome, ketogenic, low carbohydrate diet, carbohydrate-restricted diet

Insulin resistance is characterized by elevated insulin levels. The most commonly used approaches to treat conditions caused by insulin resistance, including Type 2 Diabetes (T2DM), involves medications. The management of insulin resistance with medications then typically leads to a need for “intensification of medications” to improve glycemic control (1). However, giving medications like insulin to treat insulin resistance and/or T2DM does not cure these conditions—it perpetuates or even worsens the insulin resistance. This consequence is especially likely if medication treatment leads to weight gain, because weight gain leads to a worsening of the insulin resistance. An alternative approach to the treatment of insulin resistance is to use strategies that *lower* insulin blood levels, including non-pharmacologic treatments.

Obesity Medicine is a medical subspecialty that treats T2DM by targeting underlying contributors of insulin resistance and T2DM: obesity and lifestyle. One of the lifestyle approaches used by obesity medicine specialists is a carbohydrate-restricted diet (2). Several studies have shown that a carbohydrate-restricted diet can lead to improvements and even *reversal* of T2DM (3, 4).

Because many barriers exist to the implementation of carbohydrate-restricted diets into medical practice, this Research Topic invited articles related to the use of carbohydrate-restriction for T2DM. In this Research Topic, the paper topics range from theoretical to practical aspects of the use of carbohydrate restriction to *reverse* insulin resistance and T2DM.

In the first article, Westman gives an often overlooked perspective on how relatively little glucose resides in the human bloodstream at any given moment, and emphasizes the importance of dietary carbohydrate as a major factor in addressing T2DM. Wheatley et al. review many of the studies regarding carbohydrate restriction and the treatment of T2DM, with a focus also on some of the common concerns that still exist.

Then, several clinical series are presented that demonstrate the efficacy of carbohydrate-restricted diets in clinical settings. Wolver et al. estimate that 70–90% of patients were no longer taking insulin after 1 year, depending on the level of adherence—AND had improved glycemic control and weight loss. Gavidia and Kalayjian address the dogma that improvement in diabetes is a result of weight loss, not diet change. They report three cases of a substantial reduction in hemoglobin A1C without clinically meaningful weight loss.

Using carbohydrate-restricted diets can lower the blood glucose dramatically on the 1st day of the dietary change, so the reduction of medication (“de-prescribing”) is often needed on the 1st day of the dietary change. Cucuzzella et al. give advice on how to de-prescribe medications when using a carbohydrate-restricted diet. Finally, Griauzde et al. address the challenges that exist in translating the evidence-base of carbohydrate-restricted eating into clinical practice.

Carbohydrate-restriction provide an attractive approach to treat conditions caused by insulin resistance without the need for medications. Carbohydrate-restriction reverses insulin resistance by lowering the blood glucose and insulin levels, and by a loss of fat mass. While no prevention studies are available yet, it is logical to hypothesize that using carbohydrate-restriction may also be useful for the *prevention* of overweight, prediabetes, metabolic syndrome, and T2DM. It makes sense to now study the use of carbohydrate-restricted diets for disease prevention, especially in children.

## Author Contributions

The author confirms being the sole contributor of this work and has approved it for publication.

## Conflict of Interest

EW receives royalties from the sale of books related to low carbohydrate diets and is founder of a company based on the principles of carbohdyrate-restriction.

## Publisher's Note

All claims expressed in this article are solely those of the authors and do not necessarily represent those of their affiliated organizations, or those of the publisher, the editors and the reviewers. Any product that may be evaluated in this article, or claim that may be made by its manufacturer, is not guaranteed or endorsed by the publisher.
